# Supplementary material for: Peptide-based quorum sensing systems in Paenibacillus polymyxa
Source: Life Sci Alliance. 2020 Aug 6;3(10):e202000847. doi: 10.26508/lsa.202000847 (PMC7425212; doi:10.26508/lsa.202000847)
Supplement: Supplementary file 1 [file LSA-2020-00847_Supplemental_Data_1.pdf]

TPRpred analysis results for AloR receptors

Supplementary data 1

Peptide-based quorum sensing systems in Paenibacillus polymyxa

Maya Voichek, Sandra Maaß, Tobias Kroniger, Dörte Becher, Rotem Sorek

---

> PPTDRAFT_00471_AloR1

MKHTPTIRAELDRYLQQEGMSLTQFGHVAGMNRGIVSAIVTGNKSMSVNQLDLITEAMGLPEGHFYDLFIENYIIDHPPNMRRIEPLLFRC
AELDKLEAIRRVVGAIMDNLLYSPKLFEIAEELLSQERHAAALLLYEGVAEAEKYQHSERLAVCQYRIFTIQVGDDQSRNHKAAILFEPYVERL
DDLEQLDALKDLANVYRSLRKWDKVDEIARVMRAKAEIQYNWKHQQKNQERNEAAKKLSRPMFVYITYADLLCASVCDARGDYQQALQ
YTYAYADLDWVRETDDDTLYWVEMYQDWARCNIYVNKLLSGDVSVLSDYVEYIAETADTMEGEKLSKLLNVMIAANQYNLDVDDVLSRF
KKDIDAFTQLPTSTDMYSQQVVPDYIAWFGYELAYYYLNRERYSDGFKQLLFAMVKSHIINNETYFINCIGLFMRFQVYATPEIKTEFSNLIEK
VWVGNVEKNRTVNCCE

The sequence name: **PPTDRAFT_00471_AloR1**
Per-protein P-value for being TPR: **4.6E-03**
Probability for being TPR: **0.02%**
Per-protein P-value for being SEL1: **5.6E-02**
Probability for being SEL1: **0.00%**

Repeat	Begin	Alignment	End	P-value
TPR	114	--SPKLFEIAEELLSQERHAAALLLYEGVAEAEKYQ--	147	2.7e-03
TPR	191	--LDALKDLANVYRSLRKWDKVDEIARVMRAKAEIQ--	224	1.2e-03

>PPTDRAFT_00474_AloR2

RAAAVFEAFVERLDEIDQLDALKDLANVYRSLRKWDKVDEMAKKMRAKAEIQYTLKHQQNSREHTEFTDKLSRPLFVYISYADLLCASVCEA
QDDYQQALDYTYTYANLDWVMETDEDTRHWISLFKSWAEGNTYVYKLLSGDINVLHDYVEYIVATSATNENEKISKLLNVIIAANQYSIDV
DDILKRFESEINSFSEHPLSDDMYTQQVIPDYIAWFGYELAYYYLHRGSYNDGFKHLMYSMVSYHILNNETYFINCMGLFSHFREYASHETKA
SFLNLIEKVWMTNVEKNGTADHCG

The sequence name: **PPTDRAFT_00474_AloR2**
Per-protein P-value for being TPR: **7.0E-03**
Probability for being TPR: **0.01%**

Repeat	Begin	Alignment	End	P-value
TPR	19	--LDALKDLANVYRSLRKWDKVDEMAKKMRAKAEIQ--	52	7.0e-03

>PPTDRAFT_01046_AloR3

MEITPTIQAEVQTYLKRKSLTMTEFGHTIDLNVGTVSGIVTGNRSMSVHQLDSITAGMNLPPDYFYERYIEECIEECPLNWKKISPFLYRCIEL
GRLDCLQRVVILLLDNPNYLPSLFEVAENVYQDGYNEAAAYLYKKVAESEKQQHSERLAICQYRLFQIKVGQDRAVNLQAAIEFAPFVDRLD
EIEQLDALKDLANVYRSLSMWDKVYEFAHQMGQLGQLQYHLVHNSKRKGTEPRKKLSRPLFVYIVYAELLCANACDAKGNHDQALEHIRG
YADLNWVKEQDSDTLYWKRKYQQWAKINTYVNRLMSGDISVLPDYVEHIAAEKEILAELLNVLEVANRYNVDVDHILRRFESQIAAYQESP
SSDMYTQQVLTEEYVRFWYKIAKYSLNKGRYTYGFKCLLIAFEKAVTINHVLLIANCFGLFLHFKTNAAPKTQAQFHSIYEEVWEKNDQKDG
FLLGGN

The sequence name: **PPTDRAFT_01046_AloR3**
Per-protein P-value for being TPR: **2.9E-05**
Probability for being TPR: **1.19%**

Repeat	Begin	Alignment	End	P-value
TPR	114	--LPSLFEVAENVYQDGYNEAAAYLYKKVAESEKQQ--	147	6.2e-03
TPR	149	--SERLAICQYRLFQIKVGQDRAVNLQAAIEFAPFV--	182	1.5e-01
TPR	191	--LDALKDLANVYRSLSMWDKVYEFAHQMGQLGQLQ--	224	4.0e-04
TPR	340	--EVANRYNVDVDHILRRFESQIAAYQESPSSDMYT--	373	4.4e-02
TPR	382	--VRFWYKIAKYSLNKGRYTYGFKCLLIAFEKAVTI--	415	3.8e-04

>PPTDRAFT_01711_AloR4

LYNTTTTILTEIAKHMRDHDLILSDLARAAEMNPGTMSSIINGNRTLSVDQLDRITQAMGYPVGYYYERYVSEYLAEANPNWRRMSPFIHN
CARLNKLDCIHDVVNLLMDKLGYSESLFELAEELFHEKMYQAATILYENVAISEKKQYSERLAMCQYRLFHTRQGDNQEKNYEAAVQFEIYV
DRLDEIEQLDALKDLANTYRSLRKWDKVELFAKALGDKAKIQYKLEQERHPSKKENAKKPSFPLFAYWAFSHLLRAEVCEAHKDYEKALQHT
YKYGDLSWVKETDDTTLKWKRQYEEWAVGNTYLNKILAGEICVLSDYLVYISSRKDEILPALDIIVDSANRYHFDVDVILKQFEGEISSLLEQQ
KIESFYTKRFITERFMHFSKELSIYYLTKKRYSDGFKFLISCLEKSTLINNKSYIIKCMKLYESYREYASFDVKTAYRKLINEVDQDEA

The sequence name: **PPTDRAFT_01711_AloR4**
Per-protein P-value for being TPR: **7.9E-06**
Probability for being TPR: **3.04%**
Per-protein P-value for being SEL1: **8.1E-02**
Probability for being SEL1: **0.00%**

Repeat	Begin	Alignment	End	P-value
TPR	3	--NTTTTILTEIAKHMRDHDLILSDLARAAEMNPGT--	36	9.7e-03
TPR	115	--SESLFELAEELFHEKMYQAATILYENVAISEKKQ--	148	1.7e-04
SEL1	154	-AMCQYRLFHTRQGDNQEKNYEAAVQFEIYVDRLDEI-	189	3.9e-03
TPR	192	--LDALKDLANTYRSLRKWDKVELFAKALGDKAKIQ--	225	4.1e-03

>PPTDRAFT_01713_AloR5

MEIIPTIRAEVQTYLIRKSLTMTEFGHIIDLNVGTVSGIVTGNRPISVHQLDRITMGMNLPPDYFYERYIDECIEEEPLNWRRISPFLYRCVVLG
RLDCLRRVVGMLLDNPVYLPSLFEVAEDSFKKGNTEAAAFLYENVSEGEKYQYSERLAVCQYRLFTIRIGDDQEKNYDAAIQFEPYVDRLDEI
DQLEALKDLANTYRSLRKWDKVELFAKAMGDKAKIQYKLQQQRHHVHKDNSKKPNFPLFAYWAFSHLLRSQACGERKDYEKGLQHIKKY
ADLSWVTDTDEEALKWRKKYEDWAVVNTYVNKLLSGDESVLSDYVAYISARKDEILPALDIMIEAANRYHFDVDNILKQFEEEIISFLEQKKV
EGLYTQRFTTERYTHFARELAIYLLRKGKFSDGFTFLFSCLEKSAEANNRIQAIRCMRLFTHFKEHASAHTRAVYGKLIKAVNEDEE

The sequence name: **PPTDRAFT_01713_AloR5**
Per-protein P-value for being TPR: **5.4E-05**
Probability for being TPR: **0.74%**
Per-protein P-value for being SEL1: **3.9E-02**
Probability for being SEL1: **0.00%**

Repeat	Begin	Alignment	End	P-value
TPR	114	--LPSLFEVAEDSFKKGNTEAAAFLYENVSEGEKYQ--	147	1.8e-03
TPR	191	--LEALKDLANTYRSLRKWDKVELFAKAMGDKAKIQ--	224	3.6e-03
TPR	251	--AFSHLLRSQACGERKDYEKGLQHIKKYADLSWVT--	284	8.6e-03

>PPTDRAFT_01722_AloR6

MKITPTIRAELEQYLKQEGLTLSQFGQITGMNRGIISSIVTGNKSMSINQLDRITEAMGLPEGHFYDLFIENYIIDHPPNMRRIEPFLYRCAELE
KLDAIRRVVGAIMDNLLYSPKLFEIAEGLLAQGRLEAALILYEGVAEAEKYQHSERLAICQYRLFKIQIGNDQEKNFQSALQFEPFVKRLDAID
QLDALIDLANVYRSLRKWERVKEIAKDLRTLAKAQYSLEYSQKRGECKPQRPLFFYIAFSKSLIGGVYYEQGKYELALQIAYESCDLDWVKETD
EEALRWKSLFQEWGEANICLTKLMAGDTEVLPLYIDLISTNREELLPGLWNIMIVANRYKLNVDSILSQFEWEISNIHDQEGRQTNDKQNIN
DQLVGFLSELADYYLTNGQYKKGISYLLDCMRISTIINNERSILRCVRMFERCRQFVTDEAKVEYRNLVEMG

The sequence name: **PPTDRAFT_01722_AloR6**
Per-protein P-value for being TPR: **2.8E-11**
Probability for being TPR: **99.48%**
Per-protein P-value for being SEL1: **1.1E-02**
Probability for being SEL1: **0.01%**

Repeat	Begin	Alignment	End	P-value
TPR	114	--SPKLFEIAEGLLAQGRLEAALILYEGVAEAEKYQ--	147	2.6e-04
TPR	191	--LDALIDLANVYRSLRKWERVKEIAKDLRTLAKAQ--	224	1.4e-04
SEL1	222	-KAQYSLEYSQKRGECKPQRPLFFYIAFSKSLIGGVY-	257	8.5e-03
TPR	247	--AFSKSLIGGVYYEQGKYELALQIAYESCDLDWVK--	280	1.1e-03
SEL1	264	-ELALQIAYESCDLDWVKETDEEALRWKSLFQEWGEA-	299	9.7e-04
TPR	379	--VGFLSELADYYLTNGQYKKGISYLLDCMRISTII--	412	1.0e-03

>PPTDRAFT_02243_AloR7

VDDLVHSDITDLDTLVNMIKLSFSFMDGTHMIALCDQLYYHVNQKYQEWQLYKAFKKPIQPIHTKRPLVYYYGYSHLMKGMAFQKQGKYE
AARDCIEKYAELGWFNGLDPYGEAEVDYYRHAARANLYALDILSGRSEMLQEYVQFLRDNPEELLPGLLSIVEGATRHDYPLERVLESFAEQI
EDFKYFEEPVNVSYYFRFCYQLALYYIKLRQFRVALGYILQSLKLSDTLGMEHEHEFRKCTAIFEIFRSQATATQQDQYITILKGVLKDEKIDFTP
VVAQSV

The sequence name: **PPTDRAFT_02243_AloR7**
Per-protein P-value for being TPR: **1.8E-10**
Probability for being TPR: **98.46%**

Repeat	Begin	Alignment	End	P-value
TPR	73	--GYSHLMKGMAFQKQGKYEAARDCIEKYAELGWFN--	106	1.7e-06
TPR	199	--FRFCYQLALYYIKLRQFRVALGYILQSLKLSDTL--	232	1.2e-04

>PPTDRAFT_02798_AloR8

MKHTPTIRAELDRYLKQEGLNLTQFGHIAGINRGIVSGIVTGNSSMSVNQIDRITEAMGLPEGYFYDLYVENYIIDIPPNMRRIEPFLYRCAEL
DKLDAIRRVVGAIMDNLLYSPKLFDIAEELLAQERHDAALLLYESVAEVEKYQHSERLAICQYRIFTIQIDDDQSRNLRAAILFEPYVERLDEIEQ
LDALEDLANVYRSLRKWDKVDEIARKMRAKAEIQYTLKHQQSSRERTESTKKLTRPMFVYITYADLLCASVCEARGDYQQALQYTYAYTDLD
WVKETDEDTQYWISLFQHWAEGNAYVNKLMSGNTSVLLDFVEYIGTTANESEKLSKLLNIIIVANRYNLDIDGVLTKFKIDIDASTQLPTSTD
MYTQQVVPDYIALFCYEMAYYNLNRKKYSDGFKQLLSAMVKAHILNNETYFINCTGLFAHFQAYASPETREEYFKLLEKVWLDNVEKNGTV
NRCE

The sequence name: **PPTDRAFT_02798_AloR8**
Per-protein P-value for being TPR: **1.5E-04**
Probability for being TPR: **0.34%**
Per-protein P-value for being SEL1: **3.8E-02**
Probability for being SEL1: **0.00%**

Repeat	Begin	Alignment	End	P-value
TPR	114	--SPKLFDIAEELLAQERHDAALLLYESVAEVEKYQ--	147	3.0e-04
TPR	191	--LDALEDLANVYRSLRKWDKVDEIARKMRAKAEIQ--	224	3.2e-04

>PPTDRAFT_02818_AloR9

MKHTPTIRAELDRYLQQEGLSMTEFGHIAGMNRGAVSAIVSGNKSMSVNQLDLITEAMGLPEGHFYDLFIENYIIDHPPNMRRIEPFLFRC
AELDKLDAIRRVVGAIMDNLLYSPKLFEIAEELLVQGRHKAALLLYEGVAEAERYQHSERLAVCQYRIFTIQIGDDQSQNVRVAAVFEAFVER
LDEIEQLDALKDLANVYRSLRKWDKVDEMARQMRAKAVIQYSFVHNEQHKDGTLEKKTRGPLFGYIAYADLLCASVCEARGDYQQALQYT
YAYADLDWVKEDDEDTKHWMNLFSQWAEGNTYVYKLLSGDINVLHDYVEYIVATSETNENEKISKLLNVIIAANQYSIDVDDILKRFETLIGT
FSQQLSSDDMYTRQVIPDYIAWFGYELGFYFLHRGKYIDGFKYLMNAMVKSHIINNETYFINCMGLFVRFQAHAVPETKAEYFNLIERVWE
NNVQKSGTSNHCG

The sequence name: **PPTDRAFT_02818_AloR9**
Per-protein P-value for being TPR: **4.7E-04**
Probability for being TPR: **0.13%**
Per-protein P-value for being SEL1: **5.1E-02**
Probability for being SEL1: **0.00%**

Repeat	Begin	Alignment	End	P-value
TPR	114	--SPKLFEIAEELLVQGRHKAALLLYEGVAEAERYQ--	147	7.3e-04
TPR	191	--LDALKDLANVYRSLRKWDKVDEMARQMRAKAVIQ--	224	4.1e-04

>PPTDRAFT_03195_AloR10

MKITPTIRAELEHFLQQENLTLSQFGQITGMNRGIISSIVTGNKSMSINQLDRITEAMGLPEGHFYDLFIENYIIDTPPNMRRIEPFLFRCAELD
KLDAIRRVVGAIMDNLLYSPKLFEIAEELLSQGRHEAALILYEGVAEAERYQHSERLAVCQYRIFTIQVGDDQSRNIRAAAVFEAFVERLDEID
QLDALKDLANVYRSLRKWDKVDEIARKMKQKAKTQYLLKHQHKNRECDEYKKLSRPLFVYISYADLLCAGVYEAQGDYNQALQYTYAYADL
DWVKETDEDTKHWISLFQHWAQANVIVNKLLSGDMKVLNDYVEYIAASSDTTDQDRVTQLLNIMMTANRYQIDVNDILQRFETDVNSLL
QLPQFNDVYTQHVIPEQFARLGYELAYYYLHQGTYDDGFKYLMYSLVSYHRLNNETYFIKCLVLFERYRVYAVSETKAAYLEFIERVWIADVK
KNGAIDRRN

The sequence name: **PPTDRAFT_03195_AloR10**
Per-protein P-value for being TPR: **5.8E-08**
Probability for being TPR: **51.55%**
Per-protein P-value for being SEL1: **1.0E-02**
Probability for being SEL1: **0.01%**

Repeat	Begin	Alignment	End	P-value
TPR	114	--SPKLFEIAEELLSQGRHEAALILYEGVAEAERYQ--	147	9.7e-05
TPR	191	--LDALKDLANVYRSLRKWDKVDEIARKMKQKAKTQ--	224	2.4e-05
TPR	388	--ARLGYELAYYYLHQGTYDDGFKYLMYSLVSYHRL--	421	1.5e-03

>PPTDRAFT_04223_AloR11

LSFIKFAHSKFVLKGWFIMENTPTIRAEIVKYLKQKDLTMTEFGHIIDLNVGTISGIVTGNRSISVHQLDSITMGMNLPPDYFYERFIEECIEDFS
LNWRKISPFLYRCVELNRLDCVQRVVSMLLDNPAYPPLLFELAENSFQKGYKDTAAYLYEKIAESEKHQHSERLAICQYRLFTIGIGEDQIQNL
QIAARFEPFIDRLDEVDQLDALKDLANLYRALGQWDKVYEFAQMMRERAKLRYGMKQESHKRPSRPPFVYIAYGDLLCAYACDARGNYE
QGLKHLKAHTDLSWVKEKDSESQHWLGLFQGWAKINTYVNRLMSGDVSVLPDYVEYISGEKEIFAELLNVVEAANRYNLDIDHILDRFESHI
AAYREPKATDIYTQQVLSDEYARFWYKLAKYKLFRGEYPYGFKCLMDAFEKSVKINNVLLISNCSGLFERFRVHADPETLAQYQSMFLEVWE
RNEKKDGFLFSS

The sequence name: **PPTDRAFT_04223_AloR11**
Per-protein P-value for being TPR: **2.1E-07**
Probability for being TPR: **29.80%**

Repeat	Begin	Alignment	End	P-value
TPR	132	--PPLLFELAENSFQKGYKDTAAYLYEKIAESEKHQ--	165	1.6e-04
TPR	167	--SERLAICQYRLFTIGIGEDQIQNLQIAARFEPFI--	200	1.0e-01
TPR	209	--LDALKDLANLYRALGQWDKVYEFAQMMRERAKLR--	242	9.3e-05
TPR	393	--ARFWYKLAKYKLFRGEYPYGFKCLMDAFEKSVKI--	426	3.3e-04
TPR	435	--CSGLFERFRVHADPETLAQYQSMFLEVWERNEKK--	468	4.5e-02

>PPTDRAFT_04756_AloR12

LEKNTTIRAEIEKYIQSEGISMQCFADASKVNPGTLSGILNRNPPRPISVNQLDLITEAMGLEEGSLFEMYVDECFIHSLPHWRRLRPFLLRCSE
LNKLDCIRSVLGYLMDDLSNVPAIFETAEAMFEDGRHEASMLLYDCVIESEKYTHSERLAISYFRIFQIQIKDNQRNLKAAVQFHLYRSRLPEIY
ALEGLHMLSQVFALKMQWSEMEMYADELRELAQALYRNRNRFGDEHLELIKPLVYYYAQGHLLKASCYEFQGRYEESKQWIEGYADLSW
FEGLDEEGWKVVNLHRVYAQGNRLCIEIKMGNTSAIYEYITYLREHPDETLEGLNTLLESANRFGYFVDAELELFNQQLKRIWELAPEQWDT
HYRKHFNAFRFASFCKVYAEYYFRKGDYPKGLGQTLHCLQLSVDNSYTEHIIGCMALFEQHRNFATPEQQQLYQSLCLGVRANEKNHAYD
HSAGGYL

The sequence name: **PPTDRAFT_04756_AloR12**
Per-protein P-value for being TPR: **8.0E-10**
Probability for being TPR: **95.64%**
Per-protein P-value for being SEL1: **4.5E-02**
Probability for being SEL1: **0.00%**

Repeat	Begin	Alignment	End	P-value
TPR	116	--VPAIFETAEAMFEDGRHEASMLLYDCVIESEKYT--	149	1.0e-04
TPR	192	--LEGLHMLSQVFALKMQWSEMEMYADELRELAQAL--	225	4.1e-03
TPR	248	--AQGHLLKASCYEFQGRYEESKQWIEGYADLSWFE--	281	1.8e-03
TPR	383	--ASFCKVYAEYYFRKGDYPKGLGQTLHCLQLSVDN--	416	1.3e-03
SEL1	384	-SFCKVYAEYYFRKGDYPKGLGQTLHCLQLSVDNSYT-	419	1.1e-03

>PPTDRAFT_04976_AloR13

LENATTIRSEIEKELKLGGYTFNSFGQATGLNRGIFSAMLNGNPPKPISVRQMDLITKALNYPEDWLYDLYVDECFYDGRPHWKRVKPFLIRC
VEVGNLQCVEKVLSRLMEDLNHIPTIFALAEELYDGGKLQEAIPFYECVIENEKYQHSERLAISHYKLFRAKIGENTEINLKAAIQFEPFRFRLPE
CHQLDGLLHLTNIYSSLHKWQETITHSDELWEQSKIIYEEENKRRARKNRSYVPVKTERHLVVYYGQGLLMKAYALQKIGDYEKAKKIVAEYA

DLSWFEGLDTVGKREVEKFKVWSMGNSYTLDLLLGDTSVLPQYVNLLENNNDEILPGLVNILESANRNFFSVDSILDQFIKFIDTFKTTQDVIF
MDDYYNFNFQLAIYYRRNKRHKDAINAILQCIQTSFFLKNVESFAQSMVFFENLRALCDEVQIEIYKSIIKGVCKDEEGFSFTLAFSNSSQ

The sequence name: **PPTDRAFT_04976_AloR13**
Per-protein P-value for being TPR: **4.0E-12**
Probability for being TPR: **99.80%**

Repeat	Begin	Alignment	End	P-value
TPR	79	--GRPHWKRVKPFLIRCVEVGNLQCVEKVLSRLMED--	112	9.9e-02
TPR	116	--IPTIFALAEELYDGGKLQEAIPFYECVIENEKYQ--	149	3.0e-06
TPR	151	--SERLAISHYKLFRAKIGENTEINLKAAIQFEPFR--	184	1.4e-01
TPR	193	--LDGLLHLTNIYSSLHKWQETITHSDELWEQSKII--	226	1.5e-04
TPR	255	--GQGLLMKAYALQKIGDYEKAKKIVAEYADLSWFE--	288	8.3e-03
TPR	345	--ESANRNFFSVDSILDQFIKFIDTFKTTQDVIFMD--	378	7.8e-02
TPR	381	--YNFNFQLAIYYRRNKRHKDAINAILQCIQTSFFL--	414	5.9e-05

>PPTDRAFT_04977_AloR14

MEHTPTIRSTIETELKQRGYTFSSFSKISGINRGTFSTMLNSNPPKPISVRQMDLITKALGYPDGWLYELYIDECFHEGKGHWKRIKPFLLRCV
DLGRKDCIQKVLSRLTEDWSYVSTVFEFAEELHKVGKKNEAVPFYECVVENERYYHSERLAICQYRLFCYSLNSDLDNNRQAAIRFAPFPKNL
PENYQLDALLKLTNTNFQFYDYDMAIHYAKELEVLVTGIFWDQAQARAKNREVTRLNTERHLIVYYGQAYLMQGNSLEKQGLYEAAMKFI
PYYENLTRIEGLDEVGMIEAEKFEIWGKANRLNLQILMGDKDSLPAYVELIRDNPQEILLGLLTIIESANNYSYSVDDTLVLFAEDINKFRELRT
NISYYHTNNSLLTYSRFFLELSRYFFSKGSYTEAINNMLISLEASSQLNDKTYYIKSITLLFEKYKDHSTEEQRTEYEKAIKAVEYNTRQMTS

The sequence name: **PPTDRAFT_04977_AloR14**
Per-protein P-value for being TPR: **3.2E-08**
Probability for being TPR: **61.74%**

Repeat	Begin	Alignment	End	P-value
TPR	116	--VSTVFEFAEELHKVGKKNEAVPFYECVVENERYY--	149	1.5e-05
TPR	157	--CQYRLFCYSLNSDLDNNRQAAIRFAPFPKNLPEN--	190	2.7e-01
TPR	193	--LDALLKLTNTNFQFYDYDMAIHYAKELEVLVTGI--	226	1.8e-03
TPR	387	--SRFFLELSRYFFSKGSYTEAINNMLISLEASSQL--	420	2.8e-05
TPR	426	--YIKSITLLFEKYKDHSTEEQRTEYEKAIKAVEYN--	459	6.2e-02

>PPTDRAFT_05290_AloR15

VEEVLNQRGAIYLLSDSHALDKACELFRISYTHNDWDEANKVADYLHSLAESLYHTQLRNAADGKYETLDTKHPLVFYYAYSYLAKSIYYQNI
GQYEEARDYIMKYSEMGWFMGLDQSGHEEVERFRFLAKANLYMIDLLSGKKALLPEYVQFLHSNDEELLPGLVSITEAANLNNWDIDELLN
EFADDIEIFEGYEDRANRVYYLKLVNQLAIYYLKKKQYIFALNYILVSLQFSIGVEADADFRTLVALFETYRDLATPSQQEQYQTILLRGLHNEKG
IKLDNYGLSVS

The sequence name: **PPTDRAFT_05290_AloR15**
Per-protein P-value for being TPR: **5.3E-07**
Probability for being TPR: **18.02%**

Repeat	Begin	Alignment	End	P-value
TPR	2	--EEVLNQRGAIYLLSDSHALDKACELFRISYTHND--	35	2.6e-02
TPR	43	--ADYLHSLAESLYHTQLRNAADGKYETLDTKHPLV--	76	5.1e-03

Repeat	Begin	Alignment	End	P-value
TPR	80	--AYSYLAKSIYYQNIGQYEEARDYIMKYSEMGWFM--	113	5.2e-05
TPR	123	--VERFRFLAKANLYMIDLLSGKKALLPEYVQFLHS--	156	5.9e-01
TPR	163	--PGLVSITEAANLNNWDIDELLNEFADDIEIFEGY--	196	9.5e-02
TPR	206	--LKLVNQLAIYYLKKKQYIFALNYILVSLQFSIGV--	239	1.6e-03

>PPTDRAFT_05358_AloR16

LKETTTIRAELEQYIRREGITISKFGENTGINAGTISAIINGNRPIAMLLLDRIAAGMGLEEGSLYELYIDEFIRNTAPNWRRVRPFLHRCAELNK
LDCIKQVVEFIMDYLIYAPALFETAEELFSEGRLEAAAIIYKNVAESEKYQHSERLALCQYRLFTITVGDDQDENLWAATHFESFVERLGEADQ
LDALRDLANTYASLRRWDKVEWFAEKMAQKAKLQYEQKYGNTRKSERGKEPKGPLFMYIVYSYVLRAGVWDERGDYQKALEYVSLYSDL
SWVQEDTEEARHLKALCSNWAEANRYLYQLMSGSLDVIPQYVNYLEKNEGEILVGLHKIMVAANRYDFNVDHVLQHFEKQIEAFADQHA
KVGTYTEQMSADLYARFLAEMACYDLNKHRYSKGMKFLLESLSYSVVLNGNPVTIKCVGLFEKYRHTATSEDKEEYKNLLGEVEYIYDKKDRF
FSARI

The sequence name: **PPTDRAFT_05358_AloR16**
Per-protein P-value for being TPR: **6.2E-08**
Probability for being TPR: **50.32%**
Per-protein P-value for being SEL1: **2.4E-02**
Probability for being SEL1: **0.00%**

Repeat	Begin	Alignment	End	P-value
TPR	114	--APALFETAEELFSEGRLEAAAIIYKNVAESEKYQ--	147	1.1e-04
TPR	191	--LDALRDLANTYASLRRWDKVEWFAEKMAQKAKLQ--	224	2.4e-04
TPR	250	--VYSYVLRAGVWDERGDYQKALEYVSLYSDLSWVQ--	283	8.1e-05
